# Serum tumor-associated autoantibodies as diagnostic biomarkers for lung cancer: A systematic review and meta-analysis

**DOI:** 10.1371/journal.pone.0182117

**Published:** 2017-07-27

**Authors:** Zhen-Ming Tang, Zhou-Gui Ling, Chun-Mei Wang, Yan-Bin Wu, Jin-Liang Kong

**Affiliations:** 1 Department of Respiratory Medicine, the Fourth Affiliated Hospital of Guangxi Medical University, Liuzhou, China; 2 Department of Respiratory Medicine, the People's Hospital of Shenzhen Guangming New District, Shenzhen, China; 3 Institute of Respiratory Diseases, the First Affiliated Hospital of Guangxi Medical University, Nanning, China; Istituto di Ricovero e Cura a Carattere Scientifico Centro di Riferimento Oncologico della Basilicata, ITALY

## Abstract

**Objective:**

We performed a comprehensive review and meta-analysis to evaluate the diagnostic values of serum single and multiplex tumor-associated autoantibodies (TAAbs) in patients with lung cancer (LC).

**Methods:**

We searched the MEDLINE and EMBASE databases for relevant studies investigating serum TAAbs for the diagnosis of LC. The primary outcomes included sensitivity, specificity and accuracy of the test.

**Results:**

The systematic review and meta-analysis included 31 articles with single autoantibody and 39 with multiplex autoantibodies. Enzyme-linked immunosorbent assay (ELISA) was the most common detection method. For the diagnosis of patients with all stages and early-stage LC, different single or combinations of TAAbs demonstrated different diagnostic values. Although individual TAAbs showed low diagnostic sensitivity, the combination of multiplex autoantibodies offered relatively high sensitivity. For the meta-analysis of a same panel of autoantibodies in patients at all stages of LC, the pooled results of the panel of 6 TAAbs (p53, NY-ESO-1, CAGE, GBU4-5, Annexin 1 and SOX2) were: sensitivity 38% (95% CI 0.35–0.40), specificity 89% (95% CI 0.86–0.91), diagnostic accuracy 65.9% (range 62.5–81.8%), AUC 0.52 (0.48–0.57), while the summary estimates of 7 TAAbs (p53, CAGE, NY-ESO-1, GBU4-5, SOX2, MAGE A4 and Hu-D) were: sensitivity 47% (95% CI 0.34–0.60), specificity 90% (95% CI 0.89–0.92), diagnostic accuracy 78.4% (range 67.5–88.8%), AUC 0.90 (0.87–0.93). For the meta-analysis of the same panel of autoantibodies in patients at early-stage of LC, the sensitivities of both panels of 7 TAAbs and 6 TAAbs were 40% and 29.7%, while their specificities were 91% and 87%, respectively.

**Conclusions:**

Serum single or combinations of multiplex autoantibodies can be used as a tool for the diagnosis of LC patients at all stages or early-stage, but the combination of multiplex autoantibodies shows a higher detection capacity; the diagnostic value of the panel of 7 TAAbs is higher than the panel of 6 TAAbs, which may be used as potential biomarkers for the early detection of LC.

## Introduction

LC is the most common malignant tumor and the leading cause of cancer death for both sexes worldwide [1,2]. In 2015, the American Cancer Society estimated that LC was responsible for 158,040 deaths, accounting for approximately 26.8% of all deaths from cancer [3]. The average 5-year survival of LC patients is only 17%; in most patients, LC is usually advanced at the time of diagnosis, with 5-year survival rates as low as only 4% [3]. Therefore, early detection and immediate initiation of treatment are regarded as the mainstay to reduce the mortality of LC and improve the 5-year survival rate to 70–80% [4, 5]. However, because only 16% of LC patients are diagnosed at stage I [6], the detection of early stage LC patients represents a critical and challenging need in the management of this deadly disease. At present, few early detection tests or acceptable screening methods for this disease are available. Although low-dose spiral computed tomography (LDCT) has been shown to be highly sensitive for the early detection of small lung nodules and has led to a 20% reduction in LC mortality [7]. However, LDCT presents several limitations, including a high false-positive rate (as high as 50% in prevalence), repeated radiation exposure and substantial costs, which limit its widespread application as a screening procedure [8–10]. Therefore, it is necessary to develop more effective, non-invasive methods for the screening and early diagnosis of LC.

Current research efforts aim to identify the best potential and cost-effective blood biomarkers for the early detection of LC. A valid biomarker could provide additional evidence as to whether a suspicious, screening-detected nodule was malignant or not, thereby reducing the number of false positives at surgery or surgical biopsy [11]. Present diagnostic blood tests focus on detecting tumor-associated antigen (TAA) markers such as carcinoembryonic antigen (CEA), chromogranin, neuron-specific enolase, carbohydrate antigen (CA) 125, and CA19-9, which show an increased positivity at advanced stages [12] but are rarely used as early biomarkers because of their low sensitivity and specificity. However, blood tests of serum tumor-associated autoantibodies (TAAbs) against overexpressed, mutated, misfolded, or aberrant autologous cellular antigens produced by cancer cells [11,13], may identify individuals with early lung cancer and distinguish high risk smokers with benign nodules from those with lung cancer. Autoantibodies to TAAs may persist in the circulating blood longer than the antigens themselves, and may be more easily detected and have the potential to be highly useful diagnostic markers in a variety of cancers, including LC. In the blood of patients who develop lung cancer, the circulating autoantibodies have been found up to 5 years before CT was able to identify the tumor [14].

Over the years, evidence has demonstrated the potential diagnostic values of autoantibodies and their application as biomarkers for LC. Moreover, a panel of assays for autoantibodies with various TAA specificities can effectively detect LC because of the heterogeneity of single antigen expression [15]. Two recent reviews [11,16] have reported that panels of autoantibodies could be used as blood biomarkers to diagnose early LC or distinguish benign from malignant nodules; however, no meta-analysis was performed to evaluate the diagnostic accuracy of multiplex autoantibodies in these analyses. Furthermore, many relevant studies in this field have been recently published. Hence, we conducted a comprehensive review and meta-analysis to assess the diagnostic values of serum single and multiplex autoantibodies in the patients with lung cancer, especially for the early detection of LC.

## Methods

### Search strategy

We searched relevant studies from the MEDLINE and EMBASE databases until September 26, 2016. The following combination of search terms was used to retrieve articles: (lung neoplasms OR lung carcinoma OR lung cancer OR lung tumor) AND (autoantibodies OR antibodies OR immunoglobulin) AND (sensitivity OR specificity OR accuracy) in the Title/Abstract. Related or additional articles were also identified by manually searching the references cited in the articles. This process was performed repeatedly until no additional articles could be identified. Although no language restrictions were imposed initially, the full-text review and final analysis were limited to articles published in English or Chinese. If evidence showed that some publications were associated with the same study (e.g., two or more articles with the same authors, institutions, or period of study), we only selected the most recent article and the best-quality study. Two authors (ZMT and ZGL) independently determined the study eligibility while screening the citations. Disagreements were resolved by discussion and consensus.

### Study selection

We initially read the titles and abstract and obtained the full texts of the selected studies that met the eligibility criteria. To be included in our systematic review and meta-analysis, studies had to satisfy the following criteria: 1) the participants were evaluated for the presence of serum autoantibodies or antibodies; 2) the studies provided both the sensitivity and specificity of the levels of mixed autoantibodies for the diagnosis of lung cancer; and 3) studies included cancer-free patients or normal populations as a control group. Studies were excluded if they were: 1) conference abstracts and letters to journal editors; 2) reviews, meta-analyses, or proceedings; 3) studies concerning the function of autoantibodies in animal models; and 4) studies with small sample sizes (n<10) to avoid selection bias.

### Data extraction and quality assessment

Two reviewers (CMW and JLK) independently extracted the following information from all eligible articles: first author, year of publication, location, TAAs corresponding to autoantibodies, number of patients (including early-stage patients), test method, cut-off value or area under the curve (AUC), and evaluation indexes (sensitivity, specificity and accuracy). We computed manually the accuracy using the equation (diagnostic accuracy = 100×(number of true-positive + number of true negative)/total number of instances). We also computed the sensitivity and/or specificity for studies that did not report these estimates but provided sufficient information for their derivation. The extracted data were confirmed by another author (YBW).

Two independent researchers assessed the quality of the methodology of the included studies according to a new 11-item quality appraisal tool for studies of diagnostic reliability (QAREL, maximum score 11) [17], each item being assessed as “yes” or “no” or “unclear”, and certain items being rated as ‘not applicable’. When differences in scoring existed, a consensus was reached.

### Statistical analysis

The most frequently studied panel of TAAbs was selected as the subject of meta-analysis, which was performed using the Stata/SE 12.0 software (Stata Corp, College Station, Texas, USA). The pooled sensitivity and specificity forest plots were used to evaluate the diagnostic value of the same panel of autoantibodies, and the threshold effect was assessed using a summary receiver operating characteristic curve (SROC). The heterogeneity of the included studies was evaluated using an I^2^ statistic, which is a quantitative measure of inconsistencies across studies. Studies with an I^2^ statistic between 25 and 50% were considered to have low heterogeneity, whereas studies with an I^2^ statistic between 50 and 75% were considered to have moderate heterogeneity, and those with an I^2^ statistic >75% were considered to have high heterogeneity [18]. If homogeneity was present, fixed- and random-effect models provided similar results; when substantial heterogeneity of the individuals (I^2^ > 50%) was observed, a random-effect model only was used [19]. If heterogeneity was present, we performed a sensitivity analysis by omitting one study at a time to further explore the heterogeneity. If more than 10 studies were included in the meta-analysis, a funnel plot and Egger test were used to assess the publication bias.

## Results

### Study identification and selection

**Fig 1:**
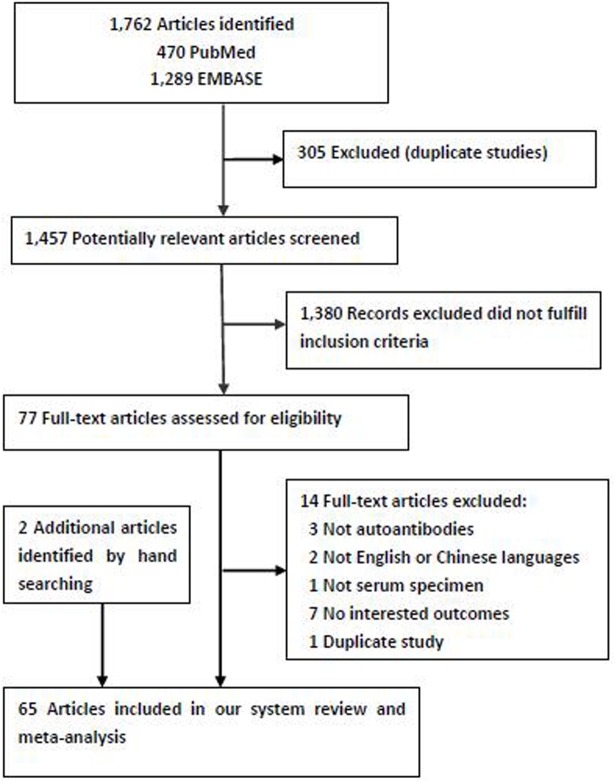
Flow diagram showing the inclusion and exclusion of studies.

A total of 1,762 potentially relevant publications were identified by the initial independent search, and 305 articles were excluded because of duplication. Overall, 1,380 publications that did not meet the inclusion criteria were excluded based on the titles and abstracts. Among the remaining 77 full-text articles, 7 were excluded because no outcomes of interest were reported [20–26], 3 were excluded because the participants were not evaluated for serum autoantibodies [27–29], 2 were excluded because it was neither in English or Chinese [30,31]. One article was excluded because the autoantibody was not performed in the serum [32], and another one was excluded because of duplicate data [33]. Two additional articles were identified by manual search [34,35]. Finally, 65 articles were included in the present system review and meta-analysis [13,14,34–96], including 31 articles with single autoantibody and 39 with multiplex autoantibodies (5 articles were related to the single and multiplex autoantibodies). The selection process is shown in Fig 1.

### Characteristics of the study populations with single and multiplex autoantibodies

For the studies with single autoantibody, the 31 articles (with 38 tests) included participants from 8 countries (Table 1). The most studied populations were Chinese [35,45,65,75,79,85,87–92,94] and Japanese [68,77,82,83,89,96], followed by American [38,71–73], Italian [78,80,93], and German [76,95].The earliest study was from 1985, and anti-CSLEX1 antibody was the first tumor-associated autoantibody to be reported in patients with LC. The sample size of the included trials ranged from 28 to 813 individuals.

**Table 1:** Studies investigating the single autoantibody.

Reference No.	Antigen	Author/ Year	Location	LC patients (AS/ES), No	Control patients (BD/NH), No	Detection method	Cut-off value	AUC	Se (%) of AS	Se (%) of ES	Sp (%) (BD/NH)	Accuracy (%), AS/ES	QAREL
35	ChgA	Qi/2015	China	168/168	97	SAM	NR	0.688	47.6	47.6	80	58.7/58.7	5
38	HSP70	Zhong/2003	USA	49	40	ELISA	NR	0.731	74	NR	73	73.0	5
38	HSP90	Zhong/2003	USA	49	40	ELISA	NR	0.602	59	NR	58	76.4	5
45	TIM	Zhang/2009	China	61	NH59	ELISA	0.221	0.790	65.6	NR	84.7	75	5
45	PRDX6	Zhang/2009	China	84/35	71(12/59)	ELISA	0.151	NR	70.5	NR	62.7	66.7	5
65	NY-ESO-1	Yang/2015	China	57/43	47	ELISA	NR	0.619	37.2	30.2	91.7	61.5/62.2	7
65	NSE	Yang/2015	China	57/43	47	ELISA	NR	0.773	48.3	NR	90.9	65.4	7
68	HP217	Okano/2016	Japan	10	18(10/8)	ELISA	0.13	NR	70	NR	72.2(60/87)	71.4	5
68	CYFRA	Okano/2016	Japan	10	18(10/8)	ELISA	3.0	NR	70	NR	100	89.3	5
71	CSLEX1	Hirota/ 1985	USA	201	612(332/280)	CBI	1:16	NR	43.8	NR	99.2(99.1/99.3)	85.5	6
72	CP	Gordon/1990	USA	22/3	74(20/54)	ELISA	0.57ug/ml	NR	86	100	87.8(80/91)	87.5/88.3	11
73	CP	Kozwich/ 1994	USA	12/3	244(106/139)	ELISA	225ng CP/ml	NR	83	100	83(83/82)	82.8/83.4	11
74	F023C5	Biggi/1991	Italy	66	8(BD)	Immuno scintigraphy	NR	NR	90	NR	45	85	5
75	3C_9_Ag	Bai/1994	China	102	172(76/96)	ELISA	43%	NR	64.7	NR	93	82.5	5
75	WLA-Ag_1_	Bai/1994	China	98	103(49/54)	ELISA	38%	NR	50	NR	95.1	73.1	5
76	NSE	Ebert/1998	Germany	50	NH98	EIA II	12.3 ng/ml	NR	78	NR	95	89.3	
77	p53	Segawa/1998	Japan	52/17	NH:63	ELISA	7.2	NR	46.1	NR	95	73.0	5
78	p53	Cioffi/2001	Italy	109/21	130(80/50)	ELISA	≥2.3 times of control	NR	32.1	42.9	100	69/92.1	5
79	p53 IgG	Zhang/2014	China	271	226	ELISA	NR	0.57	90.4	NR	19.7	58.2	5
80	TLP	Tarro/2002	Italy	ES20	25	ELISA	NR	NR	NR	88	89.9	89.0	5
81	Recoverin	Bazhin/ 2004	Rusia	143	136(86/50)	WB	NR	NR	20	NR	98	56.6	5
82	α-enolase	He/2007	Japan	94/31	NH60	ELISA	Mean+2SD	NR	27.7	NR	98.3	55.2	5
83	Nectin-4	Takano/ 2009	Japan	164/24	NH131	ELISA	1.0ng /ml	NR	53.7	25	97.7	73.2	5
84	α-crystallin	Cherneva/ 2010	England	51	52	ELISA	0.317	0.712	62	NR	72	67.0	5
85	DKK1	Yao/2010	China	93/38	NH87	ELISA	1.38	NR	62	65.8	84	64.0/78.4	5
86	SOX2	Maddison/ 2010	UK	212	NH212	ELISA	Mean+3SD	NR	33	NR	97	65	5
87	Survivin	Ma/2010	China	215/44	109(20/89)	ELISA	Mean+2SD:0.657	NR	19.5	24.1	88.9	42.9	5
88	ABCC3	Liu/2012	China	275	226	ELISA	1.53/1.58(IgG/IgA)	NR	18.1/18.0 (F/M)	NR	>95	53.1	6
89	CAXII	Kobayashi/ 2012	Japan	70	30	Dot blot analysis	NR	0.794	82.9	NR	70.0	79	5
90	IGFBP-2	Zhang/2013	China	190	104(31/71)	ELISA	1,264.306ng/ml	0.677	73.2	NR	60.6	68.7	5
91	CD25 IgG	Ye/2013	China	260/166	NH226	ELISA	NR	0.70	35	32	>90	33.6/65.3	6
92	MUC1	He/2013	China	48	27(7/20)	ELISA	1.98ug/L	NR	62.5	NR	100	76	5
93	LGALS3BP	Grassadonia/2013	Italy	13	54	ELISA	0.99	NR	46	NR	98	88.1	5
79	p16 IgG	Zhang/2014	China	271	226	ELISA	NR	0.57	19.7	NR	90.4	51.8	5
94	ANXA1	Wang/2014	China	272	NH227	ELISA	NR	0.64	23.7	NR	90.3	54.0	5
94	DDX53	Wang/2014	China	272	227	ELISA	NR	0.52	13.8	NR	90.3	48.6	5
95	TPTE	Kuemmel/ 2015	Germany	307	47	ELISA	0.0305 (ROC)	NR	52	NR	72	54.6	5
96	CANX	Kobayashi/ 2015	Japan	195/116	100	RPPA	2.49	0.980	99	NR	96.9	98.3	5

LC = lung cancer; AS/ES = all-stage/early-stage; BD/NH = benign diseases/normal healthy donors; BN = benign nodule; AUC = area under the curve; Se = sensitivity; Sp = specificity; QAREL = The Quality Appraisal for Reliability Studies; ELISA = enzyme-linked immunoassay; WB = Western blotting; CBI = cell-binding inhibition assay; EIA = enzyme immunoassay; RPPA = reverse-phase protein array; NR = not reported; SAM = significance analysis of microarray; F/M = female/male; ROC = receiver operating characteristic curve.

For the studies with multiplex autoantibodies, the baseline characteristics of 39 articles (with 49 tests) are presented in Tables 2 and 3. These studies were published between 1988 and 2016. The sample size of the included trials ranged from 28 to 2,099 individuals. Among the 12 tests from 7 articles used for the meta- analysis, 8 tests were based on the same panel of 6 TAAbs and 4 tests analyzed the same panel of 7 TAAbs.

**Table 2:** Study summary of multiple autoantibodies in the systematic review.

Reference No.	Author/ Year	Location	Combination of antigens	LC patients (ES), No	Controls (BD/NH), No	Detection method	Cut-off value	AUC/ ES	Se (%) of AS	Se (%) of ES	Sp (%) /ES	Accuracy (%) /ES	QAREL
13	Yao/2012	China	NOLC1, HMMR, MALAT1 and SMOX	40(19)	NH36	ELISA	NR	0.767	47.5	63.2	97.3	71.1/85.5	8
14	Zhong/2006	USA	L1919,L1896,G2004,G1954 and G1689	ES23	NH23	Diagnostic chip	NR	0.99	NR	91.3	91.3	91.3	6
34	Farlow/2010	USA	IMPDH, phosphoglycerate mutase, ubiquillin, Annexin I, Annexin II, and HSP70-9B	117(81)	79 BD	WB	NR	0.964	94.8	NR	91.1	93.4	5
35	Qi/2015	China	ChgA peptides (Pep16 and Pep29)	168(168)	97	SAM	NR	0.688	47.6	47.6	80.0	59.4	5
36	Schepart/ 1988	USA	5E8, 5C7, and 1F10	18(3)	BD43	ELISA	0.2ug	NR	67.0	100	81.0	77.0/82.6	
37	Bai/1994	China	WLA-Ag_1_ and 3C_9_Ag	96(15)	172(96/76)	ELISA	Mean ±2SD	NR	75.0	NR	93.8	87.1	5
38	Zhong/2003	USA	HSP70 and HSP90	49(11)	NH40	ELISA	NR	0.742	78.0	NR	65.0	71.9	5
39	Koziol/2003	USA	c-myc, cyclin B1, IMP1, Koc, p53, p62, and survivin	56	NH346	ELISA	Mean ±2SD	NR	80.0	NR	90.0	88.6	5
40	Bazhin/2003	Russia	P40-p42,p36,p30,p28,p26,p14	60	NH115	WB	NR	NR	80.0	NR	91	87.7	5
41	Pereira-Faca/ 2007	USA	14-3-3 θ, Annexin 1 and PGP 9.5	ES18	19	WB	NR	0.838	NR	55.0	95.0	75.7	7
42	Chen/2007	USA	22-Autoantibodies	75	BD50	PPM	NR	0.92	85.3	NR	86.0	85.6	8
43	Leidinger/ 2008	Germany	62 phage-peptide clones	39(18)	69(29/40)	Bayes classifier	NR	0.945	83.4	NR	93.9	90.1/92.9	5
43	Leidinger/ 2008	Germany	80 phage-peptide clones	ES18	NH40	Bayes classifier	NR	0.998	NR	79.0	99.2	92.9	5
44	Chapman/ 2008	UK	p53, c-myc, HER2, NY-ESO-1, CAGE, MUC1 and GBU4-5	104(9)	NH50	ELISA	Mean+2SD or 3SD	NR	76.0	88.9	92.0	81.2/90.9	5
45	Zhang/2009	China	TIM and PRDX6	61(35)	NH59	ELISA	NR	0.79	65.5	NR	84.7	75.0	5
46	Han/2009	South Korea	AQP5,ARTN,CKB,TAF9,TGIF2 and MCM3	17	NH15	Micro- array	NR	NR	88.0	NR	80.0	84.0	10
47	Khattar/2010	USA	Phage 908, 3148, 1011,3052 and 1000	32(11)	NH30	Peptide Library	NR	0.982	90.6	NR	73.3	82.0	7
48	Wu/2010	China	Six-Phage peptide clones 72, 91, 96, 252, 286 and 290	90(21)	NH90	Bayes classifier	NR	0.956/ 0.888	92.2	92.2	92.2/85.7	92.2/86.9	6
49	Rom/2010	USA	c-myc, Cyclin A, Cyclin B1, Cyclin D1, CDK2, and survivin	22	NH36	ELISA	Mean+3SD	0.907	81.0	NR	97.0	91.4	5
50	Murray/2010	UK	GBU4-5(G1), CAGE(P1), p53(P1) and NY-ESO-1(P1),	145	146	ELISA	Mean+3SD	NR	35.0	NR	90.0	62.5	8
51	Leidinger/ 2010	Germany	1827 proteins	47(22)	106(26/80)	SVMs	NR	0.5	97.9	75.9	97/97.6	97.6/92.9	6
53	Guergova- Kuras/2011	France	C9,LRG,Hpt,ACT and CFH	301(129)	347(112/235)	ELISA	NR	0.88	77.0	NR	87.0	82.4	5
53	Guergova-Kuras/ 2011	France	C9,LRG1,Hpt,ACT and CYFRA	301(129)	347(112/235)	ELISA	NR	0.93	84.0	83.0	95/95	90.0/91.8	5
54	Chapman/ 2011	UK	p53, NY-ESO-1, HuD, CAGE, GBU4-5, Annexin 1 and SOX2	243(14)	247	ELISA	Mean+2SD	0.76	42	50	99	70.8/96.6	6
56	Macdonald/ 2012	UK	alpha enolase BirA, p53, C-BirA, cytokeratin 8 BirA, cytokeratin 20 BirA and Lmyc2	265	265	ELISA	NR	NR	49.0	NR	93.0	70.9	5
58	Izbicka/2012	USA	EGF, sCD40 ligand, IL-8, sFas, MMP-9 and PAI-1	166	NH130	SVM	NR	NR	99	NR	95	97.3	5
59	Shan/2013	China	NY-ESO-1, XAGE-1, ADAM29 and MAGEC1	120(69)	NH68	Microarray	Mean+2SD	NR	33.0	27.5	96	55.9/61.3	5
60	Pedchenko/ 2013	USA	6 selected scFvs (B6,3E,G1,P6 and J1)	ES22	21	MSD assay	NR	0.72	NR	61.0	71.0	65.8	5
62	Wang/2014	China	Imp1, p62, Koc, p53, C-myc, Cyclin B1, Survivin, and p16.	98	58	ELISA	Mean+2SD	NR	64.3	NR	86.2	72.4	5
63	Trudgen/ 2014	USA	APEX1,NOLC1,SF3A3,PXN,R-580E16 and MT-RNR2	19(5)	237	ELISA	NR	640FU	58	80.0	43	44.1/43.8	10
65	Yang/2015	China	NY-ESO-1+NSE	57(43)	47	ELISA	NR	0.83	69.1	NR	91.8	77.0	7
66	Doseeva/ 2015	USA	NY-ESO-1, CEA, CA-125 and CYFRA 21–1	190(160)	115	xMAP	6.4	0.83	72	71.2	83.0	76.0/76.0	7
67	Wang/2016	USA	TTC14,BRAF, MORC2, ACTL2B and CTAG1B	137(110)	NH127	ELISA	>98% control	NR	30.0	NR	89.0	54.5	5
67	Wang/2016	USA	KRT8,TTC14,KLF8,BRAF and TLK1	137	BN170	ELISA	>98% control	NR	33.0	NR	88.0	63.5	5
68	Okano/2016	Japan	HP217 and CYFRA	10	18(10/8)	ELISA	0.13/3.0	NR	100	NR	72.2	82.1	5
70	Dai/2016	China, USA	14-3-3,c-Myc, MDM2, NPM1, p16, p53 and cyclin B1	90(60)	NH89	ELISA	NR	0.863	68.9	NR	79.5	74.3	5
70	Dai/2016	China, USA	14-3-3,c-Myc, MDM2, NPM1, p16, p53 and cyclin B1	25(21)	NH56	ELISA	NR	0.885	76.0	NR	73.2	75.2	5

LC = lung cancer; AS/ES = all-stage/early-stage; BD/NH = benign diseases/normal healthy donors; BN = benign nodule; AUC = area under the curve; Se = sensitivity; Sp = specificity; QAREL = The Quality Appraisal for Reliability Studies; ELISA = enzyme-linked immunoassay; WB = Western blotting; PPM = Phage-peptide microarrays; FU = fluorescent unit; SVMs = Support Vector Machines; MSD = Mesa Scale Discovery; xMAP = flexible Multi-Analyte Profiling; NR = not reported; SAM = significance analysis of microarray.

**Table 3:** Study summary of a panel of autoantibodies in the meta-analysis.

Reference No.	Author/ Year	Location	Combination of auto- antibodies	LC patients (ES), No	Controls , No	Detection method	Cut-off value	AUC	Se(%) of AL	Se(%) of ES	Sp (%)	Accuracy (%),AL/ES	QAREL
50	Murray/2010	UK,USA	6 TAAbs	241	240	ELISA	Mean+3SD	NR	34	NR	91	62.5	8
50	Murray/2010	UK,USA	6 TAAbs	269	269	ELISA	Mean+3SD	NR	37	NR	90	63.6	8
52	Lam/2011	Canda, UK,USA	6 TAAbs	574(296)	802	ELISA	Mean+3SD	NR	39	29.7	87	67.0/71.6	10
55	Boyle/2011	France	6 TAAbs	145(123)	145	ELISA	Mean+3SD	0.71	36	NR	91	63.4	10
55	Boyle/2011	France	6 TAAbs	241(1)	240	ELISA	Mean+3SD	0.63	39	NR	89	64.0	5
55	Boyle/2011	France	6 TAAbs	269(139)	269	ELISA	Mean+3SD	0.64	37	NR	90	63.6	5
57	Chapman/ 2012	UK	6 TAAbs	235	266	ELISA	Mean+2SD	NR	39	NR	89	65.7	10
64	Jett /2014	UK	6 TAAbs	26	726	ELISA	Mean+2SD	NR	46	NR	83	81.8	10
	Overall			2,000(559)	2,957								
57	Chapman/ 2012	UK	7 TAAbs	235(159)	266	ELISA	Mean+2SD	NR	41	40	91	67.5/72.0	10
61	Healey/2013	UK	7 TAAbs	607(393)	1,492	ELISA	Mean+2SD	NR	66	NR	91	83.8	9
64	Jett /2014	UK	7 TAAbs	35	812	ELISA	Mean+2SD	NR	37	NR	91	88.8	10
69	Massion /2016	UK	7 TAAbs	37	129	ELISA	Mean+2SD	NR	38	NR	84	73.5	9
	Overall			914(552)	2,699								

LC = lung cancer; AS = all-stage, ES = early-stage; AUC = area under the curve; Se = sensitivity; Sp = specificity; NR = not reported; QAREL = The Quality Appraisal for Reliability Studies; ELISA = enzyme-linked immunoassay; TAAbs = tumor-associated autoantibodies; 6 TAAbs = p53, NY-ESO-1, CAGE, GBU4-5, Annexin 1 and SOX2; 7 TAAbs = p53, NY-ESO-1, CAGE, GBU4-5, SOX2, HuD and MAGE A4.

### Tumor-associated autoantibody detection methods

Whether the studies with single autoantibody or with combinations of multiple autoantibodies, the most commonly used detection method was enzyme-linked immunoassay (ELISA), with 31 out of 38 tests for single autoantibody and 33 out of 49 tests for multiple autoantibodies. Other detection methods included Western blot (WB), phage-peptide microarray, Bayes classifier and significance analysis of microarray (SAM) et al. For the commercial panel of mixed TAAbs, the technology used to detect serum TAAbs was ELISA.

To differentiate positive and negative samples, studies most commonly used the mean absorbance or level of the TAAbs in the control group plus two or three standard deviations (SDs), or the cut-off value was determined according to the receiver operating characteristic (ROC) curve.

### Quality assessment of individual studies

For the systematic review of studies of single or multiple autoantibodies, the quality of the study design and reporting diagnostic reliability of most studies was poor since only 2 out of 38 tests with single autoantibodies or 5 out of 37 tests with combinations of multiple autoantibodies had high QAREL scores (≥8) (Tables 1 and 2). The items about examiner blinding resulted in the greatest number of “no” scores. For the meta-analysis of studies of the same panels of mixed autoantibodies, however, the methodological quality of most studies was generally good because 10 of 12 tests had high QAREL scores (Table 3).

### Diagnostic value of single tumor-associated autoantibody for any stage lung cancer

In Table 1, we have listed the single TAAb in the diagnosis of lung cancer. Overall, considering the 38 tests results for 34 specific TAAbs originating from 31 articles, the sensitivities ranged from 13.8% to 99% (mean:55.2, median: 53.7%) and the specificities ranged from 19.7% to 100% (mean:84.4, median: 90.3%). However, the diagnostic sensitivity in 17 (44.7%) individual autoantibodies was lower than 50%. Three articles reported the autoantibody against p53 [78–79], with the sensitivities ranging from 32.1% to 90.4% and the specificities ranging from 19.7% to 100%; two articles reported the autoantibody against neuron-specifi c enolase (NSE), the sensitivities were 48.3% and 78%, while their specificities were 90.9% and 95%, respectively [65,76].

### Diagnostic value of multiple autoantibodies for patients at all stages of lung cancer

The diagnostic values of mixed TAAbs for all lung cancer stages are listed in Table 2. There were 33 test results for mixed TAAbs originating from 30 articles. The sensitivities ranged from 30% to 100% (mean: 70.3%, median: 77.0%), the specificities ranged from 43% to 97.3% (mean: 86.3%, median: 90.5%), and the accuracy ranged from 44.1% to 97.6% (mean: 77.7%, median: 81.2%). In three articles, both of the sensitivity and specificity of combinations of multiplex autoantibodies were greater than 90%, which included group 1 (six-phage peptide clones 72, 91, 96, 252, 286 and 290) [48], group 2 (1827 proteins) [51] and group 3 (EGF, sCD40 ligand, IL-8, sFas, MMP-9 and PAI-1) [58]. Sixteen out of 33 tests had the diagnostic accuracy >80%.

### Meta-analysis of the same panel of autoantibodies for any stage lung cancer

**Fig 2:**
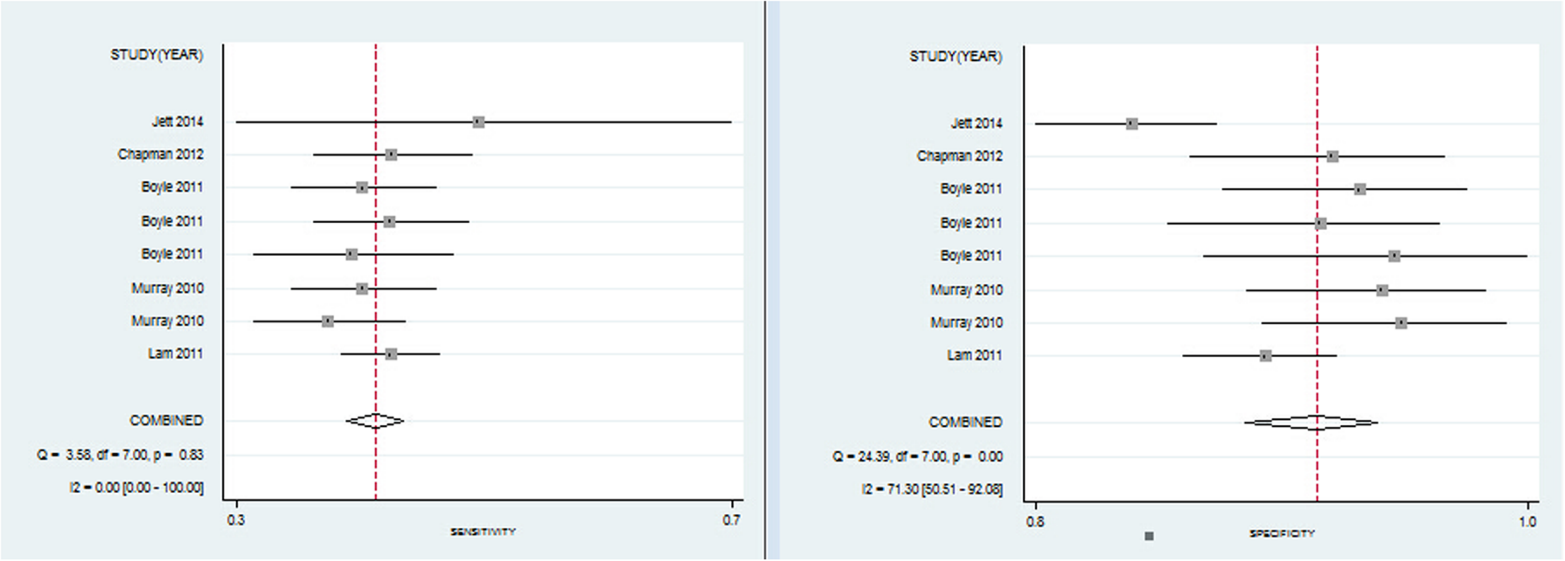
Forest plot of estimates of the panel of 6 TAAbs for sensitivity (left) and specificity (right) for diagnosing lung cancer.

**Fig 3:**
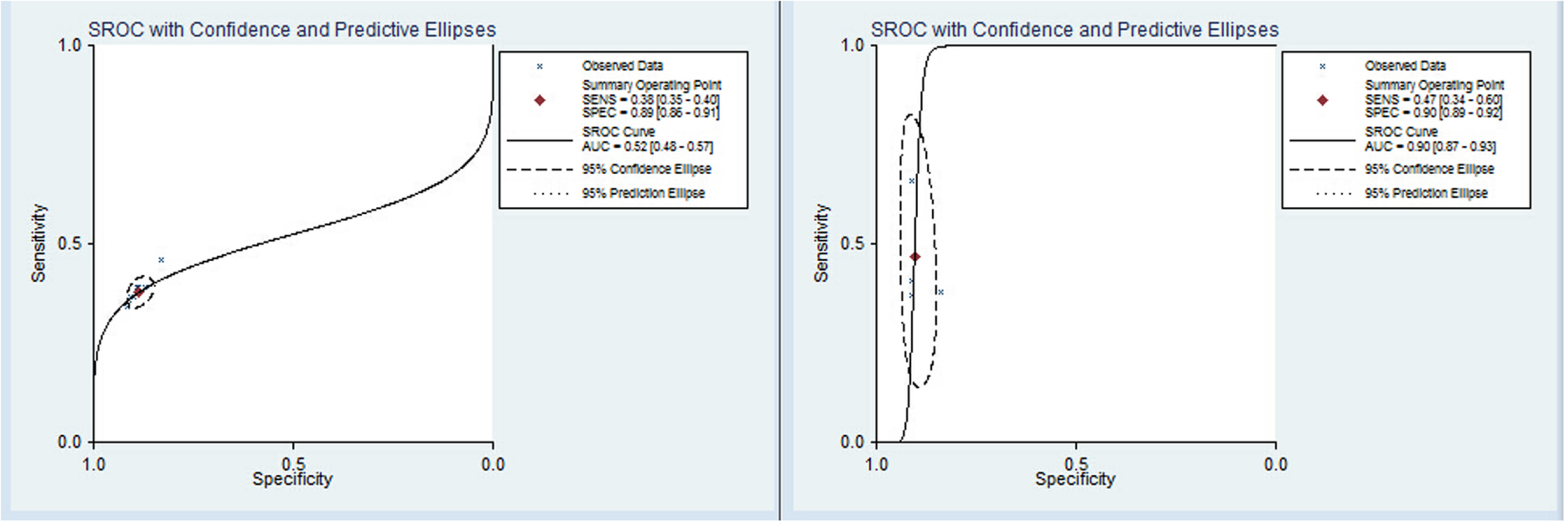
Summary receiver operating characteristic curves (SROC) for the panel of 6 TAAbs (left) and 7 TAAbs (right) for diagnosing lung cancer.

Eight tests with the same panel of 6 TAAbs (p53, NY-ESO-1, CAGE, GBU4-5, Annexin 1 and SOX2) were selected for meta-analysis. These studies were published between 2010 and 2014. The sample size of the included studies ranged from 281 to 1,376 individuals (total 4,957). The pooled estimate of sensitivity and specificity of this analysis was 38% (range 34–46%, 95% CI 0.35–0.40) and 89% (range 83%-91%, 95% CI 0.86 to 0.91), respectively (Fig 2). The diagnostic accuracy ranged from 62.5% to 81.8% (mean: 65.9%) (Table 3), while the area under curve (AUC) was 0.52 (0.48–0.57) (Fig 3-left), indicating a relative low level of overall diagnostic accuracy with the panel of 6 TAAbs. The pooled specificity of the heterogeneity test indicated that there was a moderate heterogeneity between studies (Q = 136.08, I^2^ = 94.86%, P = 0.00). Subsequently, we performed sensitivity analyses to explore potential sources of heterogeneity. The exclusion of the trial conducted by Jett and colleagues [64] resolved the heterogeneity, but did not change the pooled results (sensitivity 37%, 95% CI 0.35–0.40; specificity 89%, 95% CI 0.88–0.91; *P* for heterogeneity = 0.50, I^2^ = 0%; AUC = 0.55).

**Fig 4:**
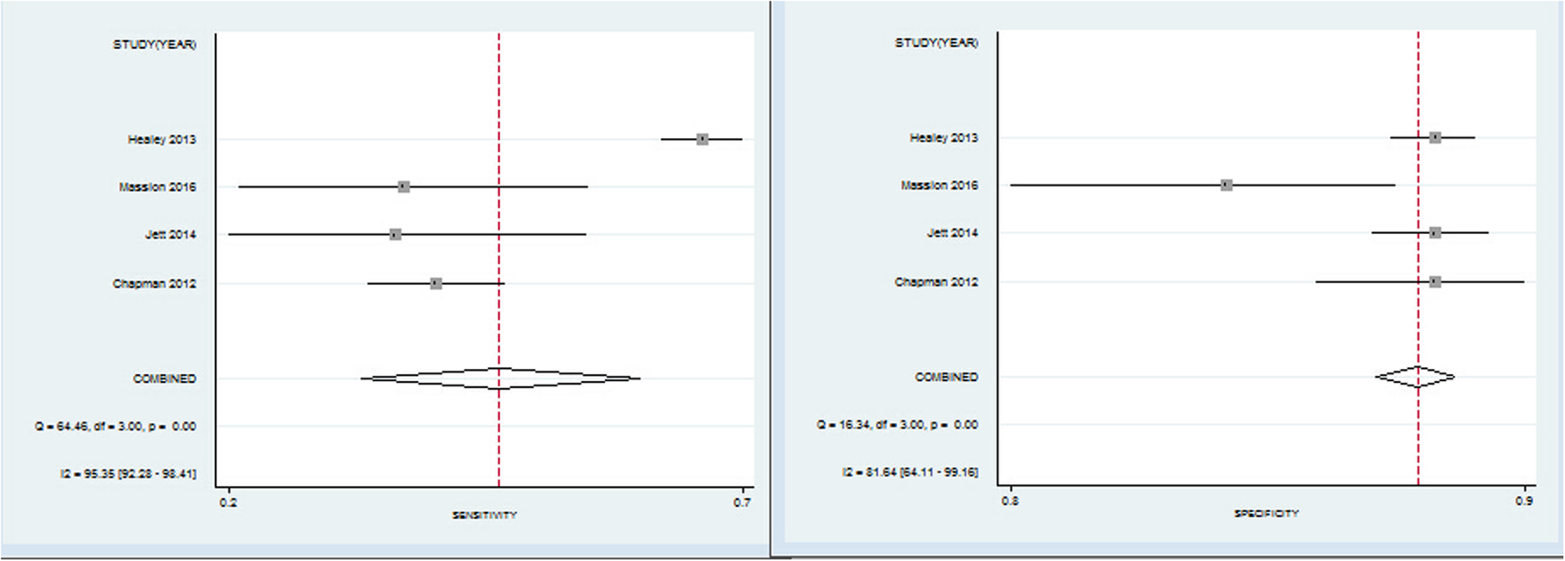
Forest plot of estimates of the panel of 7 TAAbs for sensitivity (left) and specificity (right) for diagnosing lung cancer.

Four studies that included 3,613 patients (cancer patients/controls = 914/2,699) explored the diagnostic value of the panel of 7 TAAbs (p53, CAGE, NY-ESO-1, GBU4-5, SOX2, MAGE A4 and Hu-D). The pooled estimates of this test were: sensitivity 47% (range 37–66%, 95% CI 0.34–0.60), specificity 90% (range 84%-91%, 95% CI 0.89–0.92), diagnostic accuracy 78.4% (range 67.5–88.8%), respectively, with P = 0.000 indicating a significant heterogeneity between studies. In addition, the overall AUC was 0.90 (0.87–0.93), indicating a moderate diagnostic accuracy with the panel of 7 TAAbs (Fig 3-right, Fig 4).

### Diagnostic value of single or multiple autoantibodies for early stage lung cancer

For single TAAb in the diagnosis of early -stage lung cancer, there were 10 specific TAAbs (including CP, p53, TLP, Nectin-4, DKK1 and Survivin) originating from 10 articles for the analysis, with the sensitivities ranged from 24.1% to 100%, the specificities ranged from 24.1% to 97.7% and the accuracy ranging from 58.7 to 92.1% (mean 73.4, median 75.8) (Table 1). Both of articles reported the sensitivity of CP was 100%, but the sample size was small (both with only 3 early stage LC patients) [72,73].

For the panel of mixed TAAbs in detecting early-stage lung cancer patients, 15 studies involving 2,239 patients (700 patients in the early stage lung cancer group and 1,539 in the control group) were included in our analysis. The results showed that the sensitivities ranged from 27.5 to 100% (mean: 71.1%, median: 71.2%), the specificities ranged from 43.8% to 99.2% (mean: 87.1%, median: 91.3%) and the accuracy ranged from 43.8% to 96.6% (mean: 79.6%, median: 82.6%) for the diagnosis of early-stage lung cancer(Table 2). Our data demonstrated that different combinations of multiple autoantibodies have different diagnostic values for detecting early-stage lung cancer.

For the commercial panel of mixed TAAbs for the diagnosis early-stage lung cancer, the single study reported the sensitivity, specificity and accuracy of 29.7%, 87.0% and 71.6% in the panel of 6 TAAbs [52] and 40%, 91% and 72.0% in the panel of 7 TAAbs [57], respectively. It appears that the diagnostic value of the panel of 7 TAAbs is higher than the panel of 6 TAAbs.

### Evaluation of publication bias

Publication bias was assessed, but the analysis of only 8 studies with 6 TAAbs or 4 publications with 7 TAAbs in the meta-analysis decreased the power of the publication bias analysis and limited the interpretability of the findings.

## Discussion

Different lung cancer patients are unlikely to respond to the same immunogenic antigens because of the histological heterogeneity of cancer. Even cancers of the same type are composed of different biological subtypes. In this study, for the first time, we performed a systematic review and meta-analysis to evaluate the diagnostic value of serum single or multiplex TAAbs for individuals with potential LC. Our results indicated that the single or different combination of multiple autoantibodies may have different diagnostic values for identifying patients at all stages or early-stage of lung cancer from healthy controls or benign diseases. Although the individual TAAbs showed low diagnostic sensitivity, the combination of multiplex autoantibodies offered relatively high sensitivity, and some panels of multiplex TAAbs could have promising sensitivity and specificity (both > 90%). In the present meta-analysis of a panel of TAAbs, our data demonstrated that a moderate diagnostic accuracy was achieved with the panel of 6 TAAbs or 7 TAAbs in the diagnosis all-stage lung cancer, given their AUCs of 0.52 and 0.90, respectively, indicating that the diagnostic value of the panel of 7 TAAbs was higher than the panel of 6 TAAbs in the diagnosis of lung cancer, especially in early-stage patients.

Two recent reviews [11,16] summarized some recent advances in blood-based lung cancer biomarkers that have the potential to be clinically useful in the near future, the authors found that only the miRNA signatures (the miR-Test for serum and the miRNA signature classifier test for plasma) and autoantibodies to TAAs are being assessed as noninvasive tests to detect lung cancer at the early stage. However, both of the reviews did not perform a meta-analysis of the same panel of autoantibodies. Our comprehensive review indicated that different single or combinations of multiple autoantibodies have different diagnostic abilities for detecting patients at all stages of LC, almost half of the diagnostic sensitivities in individual autoantibodies was lower than 50%. However, the combination of multiplex autoantibodies offered a relatively higher sensitivity than that of single autoantibody, with the sensitivities ranging from 30% to 100% (mean: 70.3%, median: 77.0%), the specificities ranging from 43% to 97.3% (mean: 86.3%, median: 90.5%), and the accuracy ranging from 44.1% to 97.6% (mean: 77.7%, median: 81.2%). Many combinations of multiplex autoantibodies were found to have promising value for detecting LC. *Wu* et al.[48] discovered autoantibody signatures to six–phage peptide clones (72, 91, 96, 252, 286 and 290) by two-step immunoscreenings and validated them in an independent set of 90 non-small cell lung cancer (NSCLC) patients and 90 matched healthy controls, 30 NSCLC patients undergoing chemotherapy, and 12 chronic obstructive pulmonary disease (COPD) patients. The six-phage peptide detector was able to discriminate between NSCLC patients and healthy controls with a sensitivity and specificity of >92%, and had similar value for detecting NSCLC at an early stage. The seroreactivity of the six-phage peptides was also significantly higher in the NSCLC patients than in those with chemotherapy and the COPD patients. Leidinger et al.[51] reported that an autoantibody profile consisting of 1827 integer intensity values ranging from 0 to 255 can discriminate LC patients from controls without any lung disease with a specificity of 97.0%, a sensitivity of 97.9%, and an accuracy of 97.6%. The classification of stage IA/IB tumors and controls yielded a specificity of 97.6%, a sensitivity of 75.9%, and an accuracy of 92.9%. Izbicka et al. [58] studied a set of autoantibodies (EGF, sCD40 ligand, IL-8, sFas, MMP-9 and PAI-1) as potential biomarkers. Mass spectrometry was used for biomarker discovery. A support vector machine (SVM) was used for data analysis. They found that the panel of autoantibodies was able to discriminate NSCLC patients from healthy controls with a sensitivity and specificity of 99% and 95%, respectively. However, the quality of study design and reporting diagnostic reliability were generally poor since the three publications had low QAREL scores (<8), and none of them were performed with the most commonly used detection methods, i.e. ELISA. Therefore, single autoantibody is seldom able to detect all LC with a high enough specificity and sensitivity, whereas the detection of combinations of multiple markers could significantly improve the diagnostic performance [13,68].

In the present meta-analysis, our results showed that the pooled sensitivities of a panel of 6 TAAbs and 7 TAAbs were 38% and 47%, respectively, and their specificities were 89% and 90%, respectively. The panel of 7 TAAbs yielded an AUC on a combined SROC curve of 0.90, indicating that its level of accuracy was higher than that of the panel of 6 TAAbs with an AUC of 0.52. Moreover, exclusion of a single study among the 6 TAAbs and sensitivity analyses did not materially alter the pooled results, which adds robustness to our main finding. However, both sensitivities were not very good, which indicates that a negative test result does not rule out lung cancer in the screening setting. The antigens of the panel of 6 TAAbs are p53, NY-ESO-1, CAGE, GBU4-5, Annexin 1 and SOX2. In brief, autoantibodies to p53 tumor suppressor gene, which is often mutated in a variety of malignancies (including in lung, colorectal and breast cancer), can be detected before the diagnosis of cancer in smokers with chronic obstructive pulmonary disease [97]. Besides expressed in prostate, breast, colorectal cancer and melanoma patients, the presence of antibodies to NY-ESO-1 were significantly elevated in NSCLC patients with an active smoking history and was more expressed in early NSCLC stages than in late stage [66,98]. CAGE has been reported in a variety of cancers, but not in normal tissues [99]. Autoantibodies to SOX2 are considered to be mainly detected in small cell lung cancer (SCLC) [100] The remaining antigens GBU4-5 and Annexin I are also expressed in lung cancer [54,55]. The panel of 7 TAAbs comprised two antigens (MAGE A4 and HuD) in addition to the other well-described cancer-associated antigens (p53, NY-ESO-1,CAGE, GBU4-5, and SOX2). It is possible that adding melanoma-associated antigen A4 (MAGE-A4) and HuD to the panel, which are known to have particular associations with lung cancer, may improve the sensitivity and optimize the test accuracy. MAGE A4 has been demonstrated to be expressed in melanomas and NSCLC patients (male gender, with a smoking history), especially in squamous cell carcinoma patients [98,100,101]. Approximately half of squamous cell carcinoma (SCC) expressed MAGE-A4 [102], and MAGE A4 has been proposed as a potential therapeutic target for immunotherapy [103]. HuD is a neuronal RNA-binding protein, and the HuD-antigen is expressed in 100% of SCLC tumor cells and over 50% of neuroblastoma cells [104]. In fact, anti-HuD autoantibody was detected only in SCLC cases with or without paraneoplastic encephalomyelitis/sensory neuronopathy (PEM/SN), but not in the sera of large cell neuroendocrine carcinoma (LCNEC) patients [105]. It means that autoantibodies to HuD could serve as a good marker for SCLC. Based on the QAREL score to assess the quality of diagnostic reliability, 10 of 12 publications in the meta-analysis had higher QAREL scores (≥8), suggesting that the overall methodological quality of most studies was good.

Searching for potential biomarkers of early-stage lung cancer in a high-risk population is urgently required, as this could have a markedly beneficial and clinically significant impact on patient survival [68]. Autoantibodies to TAAs has been shown to be present in patient blood for as much as 5 years before the presentation of clinical symptoms [14,44,106]. A wide variety of single or combinations of multiple autoantibodies have been reported, some of which may contribute to the diagnosis of early-stage lung cancer, while others are likely to have less diagnostic value. Our data demonstrated that different single or combinations of multiple autoantibodies have different diagnostic values for detecting early-stage lung cancer. For single TAAb in the diagnosis of early -stage lung cancer, the sensitivities ranged from 24.1% to 100%, the specificities ranged from 24.1% to 97.7% and the accuracy ranging from 58.7 to 92.1% (mean 73.4, median 75.8). Two articles reported the sensitivity of cancer procoagulant (CP) was 100% [72,73], which is expressed by a variety of malignant cells and may has potential role in the detection of early stage cancer, but the small sample size (both with only 3 early stage LC patients) in the two studies may cause an overestimation of the true effect.

For the combinations of mutiplex TAAbs in detecting early-stage lung cancer patients, the sensitivities ranged from 27.5% to 100%, and specificities ranged from 43.8% to 99.2%. Schepart *et al*.[36] reported a panel of three monoclonal antibodies (MAbs) (SE8, SC7, and 1F10) detected in three patients with Stage I or II squamous cell carcinoma. Both Leidinger et al. [43] and Wu et al. [48] found that 80 or 6 phage-peptide clones have a high accuracy for the diagnosis of early-stage lung cancer, with a sensitivity of 79.0% or 92.2%, respectively. In a study conducted by Chapman and colleagues [44], seven cancer-associated proteins (p53, c-myc, HER2, NY-ESO-1, CAGE, MUC1, and GBU4-5) were selected as markers of lung cancer with a sensitivity of 88.9% and specificity of 92% in patients with stage I-II NSCLC, but the sample size with only 9 early-stage LC patients makes the evidence limited. In another study conducted by the same authors [57], a different panel of 7 autoantibodies (p53, NY-ESO-1, CAGE, GBU4-5, Annexin 1, SOX2 and HuD) had a sensitivity of 50% and specificity of 99% in detecting SCLC patients. Some studies investigated other combinations of autoantibodies, for example, the panel of five monoclonal antibodies (C9, LRG, Hpt, ACT and CFH) [53], the panel of 4TAAbs (NOLC1, HMMR, MALAT1 and SMOX) [13] or the combination of NY-ESO-1 plus 3 tumor antigens (CEA, CA-125, and CYFRA 21–1) [66], to distinguish early-stage cancers from controls, and found that these different combinations of multiple autoantibodies have a high diagnostic accuracy for detecting early-stage lung cancer. However, some combinations of autoantibodies have a low sensitivity, for example, the panel of 14-3-3 θ, Annexin 1 and PGP 9.5, with a sensitivity of 55.0%; the panel of NY-ESO-1, XAGE-1, ADAM29 and MAGEC1 with a sensitivity of 27.5%, and the ChgA peptides (Pep16 and Pep29) with a sensitivity of 47.6%. Using a commercial biomarker assay of EarlyCDT-Lung test, Lam et al. [52] included 296 stageⅠ-Ⅱ NSCLC or limited SCLC patients, and found that the sensitivity, specificity and accuracy in the above-mentioned panel of 6 TAAbs were 29.7%, 87.0% and 71.6%, respectively. While Chapman al.[57] investigated the diagnostic value of 7 TAAbs in 159 early-stage patients, with a sensitivity, specificity and accuracy of 40%, 91% and 72.0%, respectively. Both of them can be detected in the early-stage lung cancer patients, with the AUCs 0.52 and 0.90, respectively, the diagnostic value of the panel of 7 TAAbs appears to be higher than the panel of 6 TAAbs.

There are some limitations to our study. First, we only searched two databases; therefore, we could not guarantee that all relevant studies were included. Second, the inclusion of studies published in English or Chinese may have resulted in publication bias. Third, the compositions of single or multiplex autoantibody combinations were very heterogeneous from study to study and various detection methods and cut-off points were used to distinguish LC patients from controls, which may have a potential impact on our results. It should be mentioned that, although blood-based autoantibodies have a great potential for use in the near future, these tests cannot yet be used as stand-alone tests, as they must be integrated with LDCT scan imaging in the screening procedure.

In summary, our study demonstrated that combinations of serum single or multiplex TAAbs may be useful biomarkers for discriminating LC patients at all stages or an early-stage from healthy controls or benign diseases, but the combination of multiplex autoantibodies shows a higher detection capacity; the diagnostic value of the panel of 7 TAAbs is higher than the panel of 6 TAAbs, which may be used as potential biomarkers for the early detection of LC. For physicians, a serum test integrated with LDCT scan imaging could be used as a screening tool to identify patients with suspected asymptomatic LC. Further study is needed to improve the sensitivity and specificity of the panel of autoantibodies according to different TAAs combinations.

## Supporting information

S1 Checklist A PRISMA checklist for this systematic review and meta-analysis. (DOC)

## Abbreviations

LC: Lung cancer
TAAbs: tumor-associated autoantibodies
LDCT: low-dose spiral computed tomography
TAA: tumor-associated antigen
AUC: under the curve
QAREL: quality appraisal tool for studies of diagnostic reliability
SROC: summary receiver operating characteristic curve
ELISA: enzyme-linked immunoassay
WB: Western blot
SCLC: small cell lung cancer
NSCLC: non-small cell lung cancer
